# A Physical Activity Reference Data-Set Recorded from Older Adults Using Body-Worn Inertial Sensors and Video Technology—The ADAPT Study Data-Set

**DOI:** 10.3390/s17030559

**Published:** 2017-03-10

**Authors:** Alan Kevin Bourke, Espen Alexander F. Ihlen, Ronny Bergquist, Per Bendik Wik, Beatrix Vereijken, Jorunn L. Helbostad

**Affiliations:** Department of Neuroscience, Faculty of Medicine, Norwegian University of Science and Technology, 7491 Trondheim, Norway; espen.ihlen@ntnu.no (E.A.F.I.); ronny.bergquist@ntnu.no (R.B.); per.b.wik@ntnu.no (P.B.W.); beatrix.vereijken@ntnu.no (B.V.); jorunn.helbostad@ntnu.no (J.L.H.)

**Keywords:** validation, activity, classification, algorithm, inertial-sensor

## Abstract

Physical activity monitoring algorithms are often developed using conditions that do not represent real-life activities, not developed using the target population, or not labelled to a high enough resolution to capture the true detail of human movement. We have designed a semi-structured supervised laboratory-based activity protocol and an unsupervised free-living activity protocol and recorded 20 older adults performing both protocols while wearing up to 12 body-worn sensors. Subjects’ movements were recorded using synchronised cameras (≥25 fps), both deployed in a laboratory environment to capture the in-lab portion of the protocol and a body-worn camera for out-of-lab activities. Video labelling of the subjects’ movements was performed by five raters using 11 different category labels. The overall level of agreement was high (percentage of agreement >90.05%, and Cohen’s Kappa, corrected kappa, Krippendorff’s alpha and Fleiss’ kappa >0.86). A total of 43.92 h of activities were recorded, including 9.52 h of in-lab and 34.41 h of out-of-lab activities. A total of 88.37% and 152.01% of planned transitions were recorded during the in-lab and out-of-lab scenarios, respectively. This study has produced the most detailed dataset to date of inertial sensor data, synchronised with high frame-rate (≥25 fps) video labelled data recorded in a free-living environment from older adults living independently. This dataset is suitable for validation of existing activity classification systems and development of new activity classification algorithms.

## 1. Introduction

The share of people aged 65 years and over, among the world’s dependents, has doubled since the mid-1960s, reaching 20% in 2015. Projections estimate that by 2050, older persons will account for 36% of people in the dependent age group worldwide [1]. With this projected shift in population demographics, increased demand will be placed on national health care services and budgets. The classification and monitoring of human physical activities, using wearable technology, can improve health assessment and monitoring systems and thus promote safer independent living and early detection of health deterioration in this population.

Recent developments in integrated circuit design and specifically Micro Electro Mechanical Systems (MEMS) technology has stimulated the advancement of ubiquitous body-worn inertial sensors, facilitating the accurate measurement of body-segment kinematics. These MEMS-based inertial sensors consist of a seismic mass suspended using supporting springs, etched into the silicon layer of miniature integrated circuits. Movement of the mass is governed by the combination of Hook’s Law and Newton’s Second Law, with the displacement of the mass measured using differential capacitance, and is thus proportional to the force applied resulting in a sensor capable of measuring acceleration due to movement and gravity. Thus, body-worn MEMS-based inertial sensors have been used to develop algorithms for both multi-location and single-location sensor systems for human physical activity and behaviour recognition [2,3]. However, even with the advances in wearable technology, there are several challenges related to accurately identifying aspects of human movement from body-worn sensors, the central issue being the lack of a high quality gold-standard dataset for development and validation of these algorithms.

The weak point in many validation and algorithm development studies is the selection and circumstances related to the performance of the physical activities recorded. Often, datasets are recorded in a laboratory setting, or in the person home-environment, with the researcher instructing the subject to perform a sequence of specific postural transitions and movements, which can lead to the movements being performed unnaturally. This behavioural change, due to an awareness of being observed, is known as the Hawthorne effect [4,5]. Another negative aspect of many studies is that young healthy subjects are recruited to perform the activities that are used in algorithm development, where the target group is older adults. It is thus essential to include a population-specific subject group during data harvesting that matches the target audience to avoid algorithm bias.

Supervised scripted protocols have traditionally been used to compile datasets of the quantity of desired activities, with the participant fully in the knowledge of what is being recorded [6,7,8,9]. This is otherwise referred to as a Standardized Protocol [10]. This type of protocol is suitable for compiling the required dataset in a balanced way, but can suffer from the previously described Hawthorne effect. A semi-structured supervised protocol, where participants are asked to perform a task while an observer is present logging the activities, requires specific postures, transitions or movements, to be performed in order to complete the task. This method will generate more representative activities and postures, for example, while a subject is seated at a table, they can be instructed to “pick-up an object from the floor” which is placed at a distance. This task will require sitting, sit-to-stand/walk and walk/stand-to-sit transitions, walking, standing and bending down. Protocols of this nature have previously been completed by, for example, Masse et al. [11,12], with 12 mobility-impaired stroke patients and Grant et al. [13] with 10 adults. Task-based protocols of this nature will thus produce more realistic activities.

Recently Lindemann et al. [10] provided recommendations for standardising validation procedures when assessing the physical activity of older persons through monitoring of body postures. Parameters to describe physical activity are related to body postures and movements, as characterised by the FITT principle, which considers that physical activity can be measured by four main components, Frequency, Intensity, Time and Type, where the Type of activity (i.e., the main body postures and movements) are formed by: lying, sitting, standing, walking and body transitions.

A fully free-living unsupervised protocol is where participants perform their daily routine in their own home environment, without a prescribed protocol or supervision from a study investigator. However, implementing a fully free-living protocol is not feasible, as people perform a wide variety of activities and in order to compile adequate data for each activity (e.g., lying), long monitoring periods would be required. A compromise is to monitor activity in a natural setting, and to request people to include certain tasks into their daily routine during a defined time period. This type of protocol will produce more representative activities and postures, since the user is not directly observed and is carrying out the protocol in their own home environment. Such protocols have been used by Bao et al. [14] where subjects completed an “obstacle course” unsupervised, consisting of a series of activities. However, subjects manually recorded the time they began and finished each task. Doherty et al. [15] used body-worn cameras to record people’s unscripted movements in their daily life. However, with a low frame-rate (one image every 1 to 3 s), the beginning and end of each posture and activity cannot be labelled to a high accuracy.

Even with improvements in protocol design, the methods of annotation of recorded datasets for the development and validation of activity classification algorithms can be improved upon. Such methods include self-report labelling [14]; direct human observation of the person’s movements labelled in real-time on paper [16]; using a portable device (e.g., touch screen tablet) or laptop [17]; a combination of video recordings and reference inertial sensor [12]; or a previously validated inertial sensor-based reference system [18] with the method of direct observation combined with live annotation, as reported in [17] and employed in [18]. This last method suffers from human error and inaccuracy due to attentive observation and a reported error of 1 to 3 s [17]. Video validation of inertial sensor-based activity monitors has previously been performed by Taylor et al. [19] who used video analysis to allocate four categories (standing, sitting, lying, and locomotion) in 1 s resolution; Capela et al. [20] who used six categories (stand, sit, lie, walk, stairs, and small moves) in 1 s resolution; and Aminian et al. [21] who used five categories and a resolution of 10 s. However, the video resolution of these recordings is insufficient to validate various daily life activity transitions, where typically higher resolutions of tens of frames per second is necessary [22].

The aim of this study is to resolve previous shortcomings by compiling a comprehensive reference dataset of representative activities from an older adult population that is suitable for the validation of existing activity classification algorithms and allows for the development of new activity classification algorithms using the harvested raw sensor data.

## 2. Materials and Methods

The aim of this study will be achieved in two steps: (1) develop and describe a comprehensive flexible semi-structured supervised task-based protocol, and a free-living unsupervised task-based protocol, where a wide range of representative activities and postures are included; and (2) compile a representative reference dataset using a population of community dwelling older adults recorded performing the developed protocols, while being monitored using high frame-rate video technology of ≥25 fps (≤0.04 s resolution) and a selection of multiple, synchronised body-worn inertial sensors.

### 2.1. Subjects

A convenience sample of 20 older adult participants was recruited from a senior citizen centre in the Trondheim area in Norway. As inclusion criteria, participants were required to: (1) be over 65 years of age; (2) be able to walk 100 m without walking aids; (3) accept oral instructions; and (4) be living independently. A total of 5 male and 15 female were recruited, ranging in age from 68 to 90 years (76.4 ± 5.6 years), body mass from 56 to 93 kg (73.7 ± 11.4 kg), and height from 1.56 to 1.81 m (1.67 ± 0.072 m). The Regional Committee on Ethics in Medical Research in Central Norway approved the trial protocol and subjects provided written informed consent.

### 2.2. Sensor Set-Up

**Figure 1:**
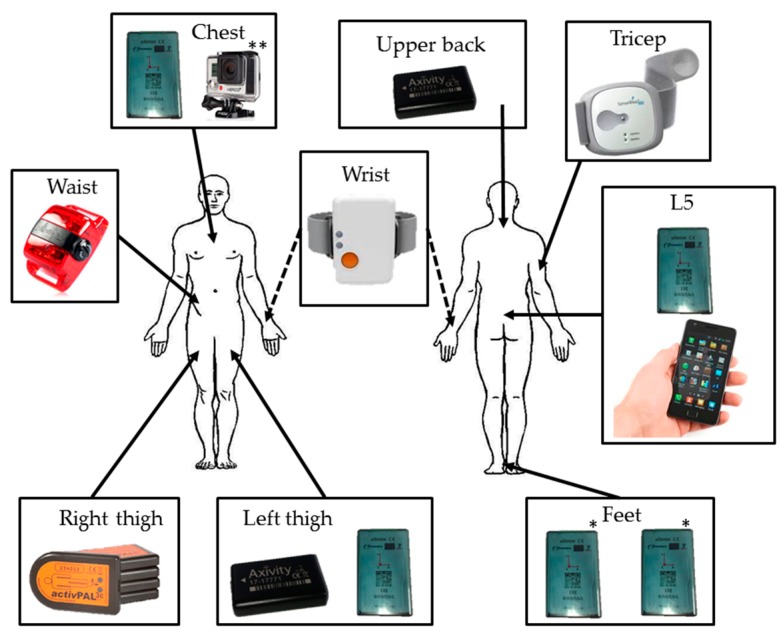
The sensors configuration, where * indicates the sensors that will only be used in the semi-structured protocol, and ** indicates the camera that will be attached for the out-of-lab activities.

The choice of sensors and body locations was motivated by the potential for algorithm development from popular activity monitoring device attachment locations [2,3] and existing large datasets recorded from independent living older adults in previous projects, where detection of falls and the assessment of fall risk was the focus (see Table 1 and Figure 1). These projects include the FARSEEING project [23], the Generation 100 project [24], the PreventIT project [25] and the HUNT population-based study [26]. Through developing accurate activity classification algorithms from different body-worn sensor locations, used by the sensors in each project, a common output can be obtained. This harmonises these datasets and allows for the development of fall-risk assessment algorithms through a common physical activity output.

### 2.3. Activity Selection

**Figure 2:**
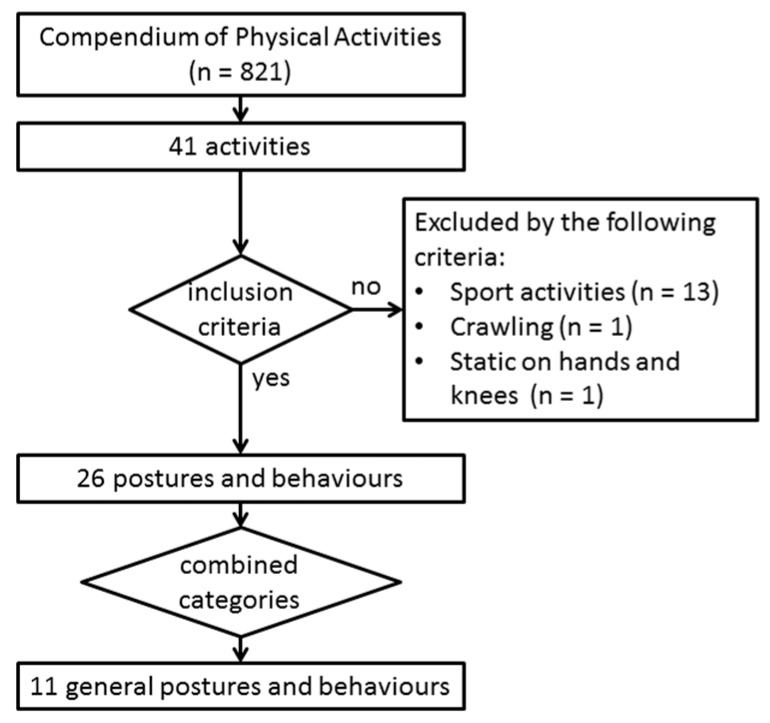
Flowchart depicting the selection of the 11 general postures and behaviours that are listed below in Table 2.

A list of activities that are commonly performed in everyday life by older adults was compiled using the following procedure (see flowchart Figure 2). First, the Compendium of Physical Activity by Ainsworth et al. [27] was consulted to identify individual postures and behaviours that occur in everyday life; Second, these postures and behaviours were combined to 41 independent categories (e.g., walking, sitting, standing, etc.); Third, activities related to sport and other confounding activities were excluded, resulting in a list of 11 individual posture and behaviours represented in Table 2 that are related to daily physical activity. Fourth, transitions between the 11 general postures and behaviours were defined, as presented in Table 3. Several transition types were not included as part of the protocol as they are either rare events (e.g., lie-to-picking and lie-to-leaning) or will not induce a meaningful transfer (e.g., picking-to-leaning, kneeling-to-picking, and kneeling-to-leaning). Two task-based protocols were then designed to collect a minimum sufficient number of the desired transitions, (1) a supervised semi-structured protocol and (2) a free-living unsupervised protocol. A more detailed breakdown of the desired quantity of general postures, transitions and behaviours for the supervised semi-structured protocol is described in Appendix A, Table A1, and the free-living protocol in Appendix B, Table A2.

### 2.4. Supervised Semi-Structured Protocol

**Figure 3:**
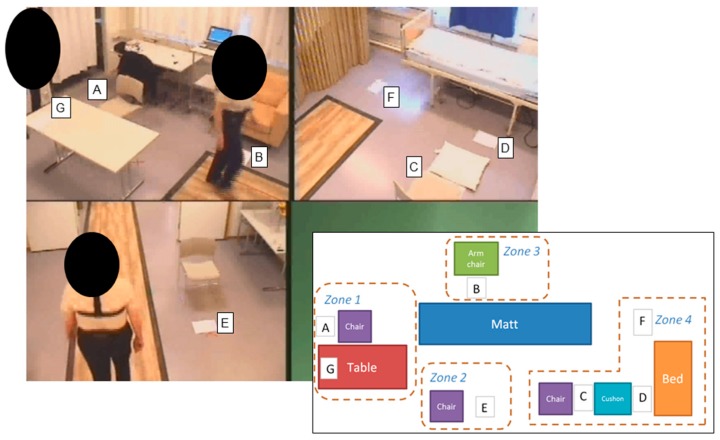
View from the in-lab cameras as a subject performs the semi-structured protocol and the floor plan of the four different activity zones used in the instructions to the participants, represented in Appendix C, Table A3.

The semi-structured protocol was performed in a smart-home environment in the Usability Laboratory at the Faculty of Medicine at the Norwegian University of Science and Technology, Trondheim, Norway. This laboratory consists of three rooms plus an observation room. The three rooms contained different types of furniture and ceiling-mounted cameras, which are monitored and controlled from the observation room (see Figure 3). MultiCam Studio and Camtasia Studio screen software was used to control and capture the camera feeds from the smart-home environment. The resulting video was recorded at 25 fps at a resolution of 768 pixels × 576 pixels in an AVI file format.

The subjects were instructed to perform the task-based protocol described in Table 4, where the instruction set is presented in Appendix C, Table A3. A synchronisation handshake was performed in view of the cameras prior to sensor attachment. The handshake consisted of a series of static and dynamic movements of the sensors which were evident in the root-sum-of-squares accelerometer signal. Through identifying the maximum correlation between the square wave outputs from the static/dynamic video data, synchronisation between the cameras and the raw sensors’ signals is achieved. The sensors were then fitted to the participants in the configuration described in Figure 1. Once all sensors were attached, the supervised semi-structured protocol was performed by the participant, guided by one of the study investigators. Prior to completion of the stair climbing tasks, a GoPro^1^ Hero3+ camera was attached to the chest of the participant using a GoPro Chesty^TM^ harness (GoPro, Inc., San Mateo, CA, USA). A second synchronisation handshake, consisting of standing, lying and jumping was performed, allowing for synchronisation between the GoPro camera, the raw sensor’s signals and the Usability Laboratory cameras. This also constituted the transition to the out-of-lab scenario. The study investigator then instructed the participant on completing the stair climbing task. Following this the sensors attached to the feet were removed and the participants were provided with a taxi and returned home to perform the free-living protocol unsupervised, see Table 5.

### 2.5. Free-Living Unsupervised Protocol

**Figure 4:**
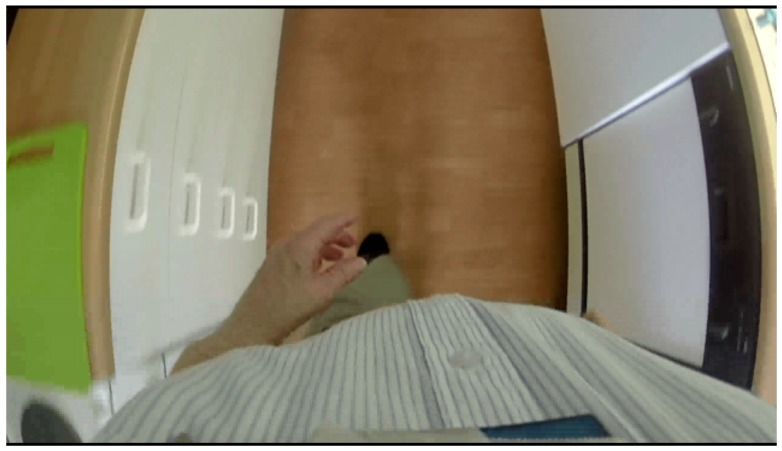
Image of the video recorded by the chest mounted Go-Pro camera. Users placed a paper-bag over the camera during bathroom breaks, which successfully obstructed the view.

The participants were instructed to perform the free-living tasks, see Table 5, in their own chosen order in their home environment. The free-living unsupervised tasks were recorded using a body-worn camera, GoPro Hero3+ camera (GoPro, Inc., San Mateo, CA, USA) with a 64 GB SanDisk Ultra XC I micro SD card, worn at the chest, attached using a harness (GoPro Chesty^TM^). Video files were recorded at 29.97 fps at 1280 pixels × 720 pixels in an MP4 format in 20-min lengths. The GoPro camera was pointed towards the feet as illustrated in Figure 4. This camera angle was chosen as it provides a view of both the subject’s lower extremities and the local environment simultaneously, thus allowing for convenient identification of the type of activity and the orientation of the body relative to the surroundings. The sensors and the GoPro camera were collected in the evening by a project co-worker, after the GoPro camera had stopped recording and the participant had removed the sensors. The camera and sensors’ data were downloaded to a computer in their respective raw data formats, using a USB interface, for later off-line data processing and analysis using MATLAB (The MathWorks Inc., Natick, MA, USA).

### 2.6. Pre-Processing and Video Annotation

**Figure 5:**
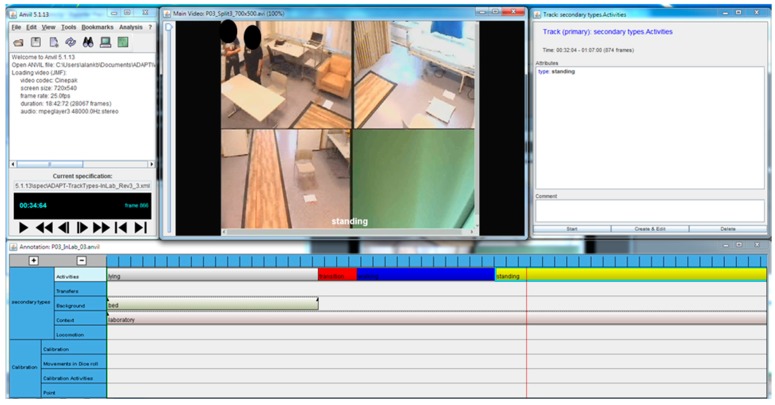
Screenprint of the Anvile software. The general postures and behaviors are visualized as boxes/rectangles on parallel tracks along a horizontal timeline. An exported frame-by-frame output was used for analysis.

The video files from the Usability Laboratory were split into files of maximum 20 min in length, using VideoPad by NCH Software (NCH Software, Inc., Greenwood Village, CO, USA) to make them compatible with the video annotation software. The videos obtained by both the wall mounted and the body-worn camera were then converted into an AVI file format with a resolution of 640 pixels × 360 pixels using a the Apple Cinepak codec, maintaining a frame rate of 25 fps and 30 fps, respectively. The videos were annotated using the Anvil software package [28]. It offers multi-layered annotation based on a user-defined coding scheme. An activity track was created where the 11 general postures and behaviours in Table 2 could be inserted (see example in Figure 5).

Four raters individually labelled the videos of in-lab activities and five labelled the out-of-lab activity videos. Raters were instructed to label the activities described in Table 2, using a set of definitions, and not allow any space between any elements in the activity track. In addition, an “undefined” category was introduced that occurred when the rater could not determine what activity the person was performing, if the camera view became blocked, or the lighting was poor. The labelling took place in a swipe-card secured PC laboratory at the Faculty of Neuroscience at St. Olav’s Hospital. One 20 min in-lab and one out-of-lab video were randomly chosen to test for the inter-rater reliability of the four and five raters, respectively. The statistics for the inter-rater reliability were Category Agreement percentage [29], Cohen’s kappa [29], corrected kappa, Krippendorff’s alpha [30] and Fleiss’s kappa.

For all video labelled data, the following statistics were recorded for both the in-lab and free-living protocols: the quantity of activities, the maximum bout length, minimum bout length, average bout length, the standard deviation, the total time and the percentage of the overall activity time.

## 3. Results

### 3.1. In-Lab Scenario

#### 3.1.1. Inter-Rater Reliability

The overall level of agreement was high for the in-lab video coding, with the percentage of agreement at 90.85% and Cohen’s kappa, corrected kappa, Krippendorff’s alpha and Fleiss’s kappa all over 0.86, see Table 6.

Fleiss’s (overall) kappa = 0.8809, kappa error = 0.0011, kappa C.I. (95%) = 0.8803, 0.8815, Perfect agreement, *z* = 794.76, *p* < 0.001 (*p* = 1.0 × 10^−21^), Reject null hypothesis: observed agreement is not accidental.

#### 3.1.2. Activities

A total of 9.521 h of in-lab activities were recorded using the semi-structured protocol (see Table 7) The activity standing was the most commonly performed activity (34.01%) followed by sitting (23.67%), transition (18.31%), walking (13.02%), shuffling (6.10%) and lying (4.09%). The activities of kneeling, picking and leaning accounted for less than 1% of all activities recorded (0.79%).

#### 3.1.3. Transitions

A total of 2640 transitions were planned for the in-lab scenario, however, 2677 transitions were recorded in total. Of the 2677 transitions recorded, 2333 were part of the protocol, while the remaining 344 were not. Thus 88.37% of planned transitions were recorded (Table 8). Out of the 22 types of transitions that were part of the protocol, 13 produced fewer transitions (range from −63.33% to −1.58% fewer), while nine produced more (range from 36.67% to 1.11%). A total of 18 transitions that were not part of the protocol were also performed.

### 3.2. Out-of-Lab Scenario

#### 3.2.1. Inter-Rater Reliability

The overall level of agreement was high for the out-of-lab video labelling, with the percentage of agreement at 90.05% with Cohen’s Kappa, corrected kappa, Krippendorff’s alpha and Fleiss’s kappa all over 0.86 (see Table 9).

Fleiss’s (overall) kappa = 0.8615, kappa error = 0.0009, kappa C.I. (95%) = 0.8611, 0.8620, Perfect agreement, *z* = 915.08, *p* < 0.001 (*p* = 1.0 × 10^−21^), Reject null hypothesis: observed agreement is not accidental.

#### 3.2.2. Activities

A total of 34.408 h of out-of-lab activities were recorded using the free-living protocol and the stair-climbing task at the end of the semi-structured protocol (see Table 10). The activity sitting was the most commonly performed activity (48.09%) followed by standing (22.17%), walking (14.22%), transition (5.12%), shuffling (4.67%), leaning (2.32%) and lying (1.33%). The activities of stair climbing, picking and kneeling accounted for 2.09% all activities recorded in the out-of-lab scenario.

#### 3.2.3. Transitions

A total of 1080 transitions were planned as part of the free-living protocol, however 3442 transitions were recorded (see Table 11). In total, 16 transitions were planned as part of the protocol, 10 produced fewer transitions (range from −100% to −4.17% fewer), with one transition not being completed at all, “lying-transition-standing”, while six produced more (range from 290% to 70% more). A total of 37 transitions that were not part of the protocol were also performed.

### 3.3. In-Lab and Out-of-Lab Activities

A total of 43.93 h of video annotation activity data were recorded (see Table 12). The activity sitting was the activity performed most often (42.8%), followed by standing (24.73%), walking (13.96%), transitions (7.98%), shuffling (4.98%) lying (1.93%) and leaning (1.84%). The activities of stair climbing, picking and kneeling account for less than 2% of the overall activity (1.78%). Considering the most common activities of sitting, standing, lying and walking (including stair ascending and stair descending) account for 84.61% of all activities recorded. However, the remaining activities of transitions, shuffling, leaning, picking and kneeling do still constitute a relevant proportion of activities (15.39%) which are often overlooked in activity classification systems.

## 4. Discussion

We have compiled a comprehensive dataset of representative activities from an independently living, older adult population recorded using two task-based protocols in a laboratory setting and a free-living setting in the participants’ home environment. This dataset is suitable for the validation of existing activity classification algorithms and will allow for the development of new activity classification algorithms using the harvested raw inertial sensor data.

A strength of the dataset is that it resulted from two protocols, a semi-structured protocol and a free-living protocol. The semi-structured protocol is designed for a laboratory setting where activities are performed under supervision; a protocol of this nature offers a compromise between achieving the desired number of planned activities and transitions with the trade-off that these are performed under supervised conditions and thus not performed as naturally as possible. The free-living protocol is designed for a person’s own home environment, where activities are performed without any supervision; a protocol of this nature prioritises the quality of the activities over the quantity of activities, ensuring that activities are performed as naturally as possible. This design makes it suitable to compare the performance of existing and new algorithms developed in an in-lab setting for an out-of-lab application.

We used video data as the gold standard for classifying activities, with labelling of the subjects’ movements performed by five raters. For both the in-lab and out-of-lab video data, the overall level of agreement was high (percentage of agreement at 90.85% and 90.05%, respectively). The Cohen’s Kappa, corrected kappa, Krippendorff’s alpha and Fleiss’ kappa were all over 0.86 for both the in-lab and out-of-lab videos for the chosen activity categories, thus demonstrating that the raters successfully labelled the video data with a high level of agreement.

A total of 43.93 h of activities were recorded, including 9.52 h of in-lab activities and 34.41 h of out-of-lab activities. Standing was the most commonly performed activity in the in-lab scenario (34.01%), ahead of sitting (23.67%), while the opposite was true for the out-of-lab scenario, with sitting performed more often in the out-of-lab scenario (48.09%) than standing (22.17%). In the in-lab scenario, transitions were performed 18.31% of the time, whereas for the out-of-lab scenario, they were performed less often (5.12%). The effect of the semi-structured protocol can be clearly seen in the increased amount of transition time in the in-lab scenario due to the intensive nature of the semi-structured protocol. 

The quantity of walking in both scenarios was approximately equal, 13.02% for the in-lab scenario and 14.22% for the out-of-lab scenario. There were more shuffling episodes in the in-lab scenario (6.1%) than in the out-of-lab scenario (4.67%), being 23.44% higher in the in-lab scenario. However, both were relatively low in both scenarios and were the fifth most common activity. Lying was much more frequent in the in-lab scenario (4.09%) than in the out-of-lab scenario (1.33%), however this can be expected as the out-of-lab recording did not include any overnight recording, and any lying activity was motivated by the influence of both protocols. The difference between the percentages of activities performed between the in-lab scenario and the out-of-lab scenario can be attributed to the influence of the semi-structured protocol and the free-living protocol. In the semi-structured protocol, participants were instructed to perform tasks which incorporated specific activities, while in the presence of a study investigator. In the out-of-lab scenario, participants were only requested to incorporate specific tasks as part of a free-living protocol and were not in the presence of a study investigator. They could thus choose to perform these tasks how they wished or not at all. In addition, the semi-structured protocol incorporated 19 different tasks to be completed three times, whereas the free-living protocol incorporated 15 tasks to be completed only once.

The activities performed during the free-living protocol are less susceptible to the Hawthorn effect [4,5] due to the unsupervised nature of the protocol; in addition, since the participants are performing the protocol in their own home and thus a familiar environment, this results in a more natural pattern of distribution of activities and performance quality. Ultimately, the application of physical activity classification algorithms is in a free-living setting. If a high accuracy can be obtained using video-validated data harvested in an in-field setting, more accurate algorithms can be developed.

The difference between the planned transitions and the recorded transitions in both protocols can be attributed to the manner in which participants were able to perform the tasks. For the semi-structured protocol the investigators planned the tasks to include specific activities. Thus, the task “Stand-sit-stand at a table” was planned to consist of stand-transition-sit-transition-stand. However, this task could have required the participant to adjust their body to position themselves to sit on the chair placed at a table. This positioning of the body, for descent into a sitting position, could have required some shuffling, which is also supported by the finding that more shuffling was performed in the in-lab setting. Thus, this task could have consisted of stand-shuffle-transition-sit-transition-shuffle-stand, for example, and thus shuffling-transition-sit and sit-transition-shuffling may have been recorded instead of stand-transition-sit and sit-transition-stand.

The analysis of the difference between the planned transitions and the recorded transitions provides an insight into modifications required that would produce a more balanced dataset of activities, which can be efficiently accumulated as part of a semi-structured protocol.

This dataset is a valuable resource for the development of new physical activity classification algorithms given the level of detail used in the annotation and the fact that a lower limit was placed on the amount of rarely observed activities, e.g., lying and transitions. Thus, if this dataset is used as part of a machine learning approach, transitions from a wide spectrum of physical activity will be used and thus more robust algorithms can be developed. However, a limitation of this study is that the dataset is not balanced. In order to create a dataset ideally suited to the development of an activity classification algorithm, using a machine learning approach, a dataset with an equal amount of each activity is preferred, thus eliminating any classification bias. This is referred to as a balanced dataset in the work by Guiry et al. [31]. Even if the dataset created here can be described as unbalanced, it does more closely reflect the proportion of activities that would occur in a real world setting, given the nature of the free-living protocol, in that the participants were unsupervised for 78.32% of the time, and were only given guidelines on which tasks to perform, not how and when. Future studies could incorporate a higher frequency of certain tasks to increase the number of transitions and activities to achieve a more balanced dataset; other techniques include using synthetic minority over-sampling [32] to artificially increase the minority classes in the dataset, or simply removing data from the dataset to create a balanced sub-set.

A strength of the current dataset is that actual older adults performed the protocols. This has not always been the case for other studies, despite being aimed at developing algorithms for classifying activities and for assessing features of movement behaviour in older adults. We included older home-dwelling adults who were independent in mobility only. Thus, this dataset is likely less suitable for analysis of algorithms for older adults that are dependent in daily life activities.

To the best of our knowledge, this study is the first to generate a dataset of inertial sensor data using a free-living protocol in an unsupervised setting, using high frame-rate video recording to label participants’ movements, producing a dataset annotated at 25 frames per second recorded from older adults. This will allow activity classification algorithms with inertial sensor data to be filtered up to 12.5 Hz (Nyquist–Shannon sampling theorem) if a window width of one frame is desired. Since the typical frequency of body motion is often below 10 Hz [33], with 99% of body motion energy contained below 15 Hz, the developed algorithms will almost entirely capture the details of human movement, thus alleviating the measurement error that is often a feature of existing activity classification devices, since these datasets are often labelled with a resolution of approximately 1 s (1 Hz) or even coarser. Thus if the measurement range of an activity classification system is within the same error range as the parameter of interest in the research question, these existing algorithms and systems will not be adequately sensitive.

Given the design of the semi-structured supervised protocol and the free-living unsupervised protocol, a wide variety of transitions, postures and activities has been generated in a way that is as natural as possible due to the task-based nature of the trial protocols. It is difficult to generate the required number of activities that a study requires in order to obtain a completely balanced dataset that consists of an adequately high number of all transitions and activities. However, in order to generate activities that are performed as naturally as possible, a study of this type should include a protocol that is as close as possible to real-life situations.

## 5. Conclusions

In conclusion, we have described the development and collection of a dataset that is suitable for validation of existing, and development of new, activity classification algorithms. The strengths of the dataset include that it consists of both a semi-structured and free-living protocol, and that it has involved older adults as participants. Furthermore, this study has produced the most detailed dataset of inertial sensor data to date, synchronised with high frame-rate (>25 fps) video-labelled data and includes a wide variety of activities recorded from older adults living independently. This dataset will be suitable for validation of existing activity classification systems and the development of new activity classification algorithms capable of classification at up to 25 Hz. Researchers are also invited to collaborate with the consortium on specific research questions and get access to the full dataset. The authors will consider each proposal for collaboration. Development and validation of algorithms using the dataset will allow for a better understanding of the accuracy of existing algorithms and has the potential to remove the measurement inaccuracy in existing academic activity classification algorithms caused by low-resolution labelling of the contributing datasets.

## Figures and Tables

**Table 1:** List of sensors and devices, attachment locations and company/institution.

Device	ActivPAL3	uSense	Axivity (AX3)	Smartphone Samsung Galaxy S3	ActiGraph (GT3X+)	Sensewear	Shimmer3
**Location**	Thigh	Thigh, L5, Chest, Feet	Thigh, Upper Back	L5	Waist	Tricep	Non-dominant wrist
**Size**	35 × 53 × 7 (mm)	67 × 42 × 10 (mm)	23 × 32.5 × 7.6 (mm)	136.6 × 70.6 × 8.6 (mm)	46 × 33 × 15 (mm)	55 × 62 × 13 (mm)	51 × 34 × 14 (mm)
**Weight**	15 g	36	11 g	133 g	19 g	45.4 g	23.6 g
**Sampling frequency**	20 Hz	100 Hz	~100 Hz (variable)	~100 Hz (variable)	100 Hz	1–8 samples/min	204.8 Hz
**Battery life/Recording time**	>8 days	72 h	Memory for 14 days continuous logging at 100 Hz	16 h	13 days @ 100 Hz	11.25 h	11.75 days @ 10 Hz/4.6 days @ 1 kHz (450 mAh)
**Sensor**	3D accelerometer	3D accelerometer, gyroscope and magnetometer	3D accelerometer	3D accelerometer, gyroscope and magnetometer	3D accelerometer	3D accelerometer, GSR, Temperature, proprietary	3D accelerometer, gyroscope and magnetometer
**Measurement range**	±2 g	±2 g, ±250°/s, ±1200 µT	±8 g	±2 g	±6 g	±2 g	±8 g, ±1000°/s, ±1900 µT
**Company/Institution**	PAL Technologies Ltd., Glasgow, UK	University of Bologna, Italy	Axivity, Bath Lane, Newcastle upon Tyne NE4 5TF, UK	Samsung Electronics Co., Ltd., Suwon, South Korea	Actigraph, 49 East Chase Street. Pensacola, FL 32502, USA	Temple Healthcare Pty Ltd., Mittagong, NSW 2575, Australia	Shimmer, DCU Alpha, Dublin 11, Ireland

**Table 2:** The general purpose categories.

General Postures and Behaviours	Category
walking	upright activity
shuffling	upright activity
stairs (ascending)	upright activity
stairs (descending)	upright activity
standing	upright posture
transition	postural transition
sitting	non-upright posture
lying	non-upright posture
leaning	non-upright posture
picking	non-upright posture
kneeling	non-upright posture

**Table 3:** Matrix of transfers between non-upright static postures and from non-upright static posture to an upright posture or upright activity. Transitions in grey were deemed unnecessary to include in the protocol as they are either rare events or will not induce a meaningful transfer.

	Standing	Sitting	Lying	Kneeling *	Object Picking	Leaning to Each Side	Stepping
**Standing**		stand-to-sit	stand-to-lie	stand-to-kneeling *	stand-to-pick off the floor	stand-to-leaning	stand-to-stepping
**Sitting**	sit-to-stand		sit-to-lie	sit-to-kneeling *	sitting pick off the floor	sit-to-leaning *	sit-to-stepping
**Lying**	lie-to-stand	lie-to-sit		lie-to-kneeling *	lie-to-picking	lie-to-leaning	lie-to-stepping
Kneeling *	kneeling-to-stand *	kneeling-to-sit *	kneeling-to-lie *		kneeling-to-picking	kneeling-to-leaning	kneeling-to-stepping *
**Object picking**	pick off the floor-to-stand	pick off the floor sitting	pick object then lie	picking-to-kneeling		picking-to-leaning	pick off the floor-to-stepping
**Leaning to each side**	leaning to each side-to-stand	lean (forward, left and right) sitting *	leaning-to-lie	leaning-to-kneeling	leaning-to-picking		leaning-to-stepping
**Stepping**	stepping-to-stand	stepping-to-sit	stepping-to-lie	stepping-to-kneeling *	pick an object-to-stepping	stepping-to leaning	

* indicates that this transfer/posture is only relevant for the in-lab protocol due to its difficult nature.

**Table 4:** The semi-structured supervised task-based protocol.

Semi-Structured Protocol
Stand-to-sit-to-stand at a table
Stand-to-sit-to-stand on a soft chair
Sit-to-kneel-to-sit
Stand-to-lie-to-stand
Lying-to-sit-to-lying
Stand-to-kneel-to-stand
Stand-to-pick an object off the floor-to-stand
Stand-to-lean to pick an object off a table forward-to-stand
Stand-to-sit, while sitting, pick an object off the floor forward-to-stand
Stand-to-sit, while sitting, pick an object off the floor right-to-stand
Stand-to-sit, while sitting, pick an object off the floor left-to-stand
Stand-to-sit at a table-to-walk-to-pick an object off the floor-to-sit-to-stand
Lying-to-walk-to-pick an object off the floor-to-lying
Sitting on a soft chair-to-walk-to-pick an object off the floor-to-walk-to-sit
Stand-to-move objects from one table to another
Stand-to-walk(normal)-to-stand
Stand-to-walk(fast)-to-stand
Stand-to-walk(slow)-to-stand
Stand-to-ascend stairs-to-stand-to-descend stairs

**Table 5:** The free-living unsupervised task-based protocol.

Free-Living Protocol
Sit at a table and write a letter/list or read
Sit on an armchair watch TV/video, or read a magazine
Sit on a low stool or toilet seat (lid down clothes on, simulation only)
Lie on a bed, clothes on
Get in and out of a car or sit on a bed
Prepare and consume a drink or food while standing
Set a table for dinner or move from one counter to another many times (up to 10) (shuffling)
Simulate unloading a washing machine for 10 s or prepare a fireplace
Pick an object off the floor then replace or tie/untie shoe laces
Climbing and descending stairs or walking up and down an inclined path
Remove clothes from washing machine and hang on clothes rack or remove rubbish from bin and dispose
Sit and prepare and eat something
Clean mirror or clean a window
Wash and dry hands
Sit at a table and read

**Table 6:** Inter-rater reliability statistics for the in-lab scenario.

Inter-Rater Reliability Statistics	Average	Maximum	Minimum
Category agreement (%)	90.85	92.34	89.03
Cohen’s kappa	0.88	0.90	0.86
Corrected kappa	0.90	0.91	0.87
Krippendorff’s alpha	0.88	0.90	0.86

**Table 7:** Summary of the in-lab activities, excluding Undefined, Static, Dynamic, Shake and Jumping.

Activities	Quantity	Maximum Bout (s)	Minimum Bout (s)	Average Bout (s)	Standard Deviation (s)	Total (s)	Total (%)
Standing	1618	296.897	0.033	7.21	16.35	11,658	34.01%
Sitting	885	267.364	0.033	9.17	20.13	8113	23.67%
transition	2692	13.233	0.234	2.33	1.40	6278	18.31%
Walking	780	23.52	0.04	5.72	3.17	4463	13.02%
Shuffling	1112	36.266	0.033	1.88	2.20	2091	6.10%
Lying	232	113.232	0.133	6.05	9.84	1403	4.09%
Kneeling	56	33.84	0.067	2.39	4.99	134	0.39%
Picking	427	2.767	0.0329	0.23	0.29	99	0.29%
Leaning	78	2.567	0.033	0.51	0.57	40	0.12%
					Total (s)	34,276.76	
					Total (h)	9.521	

**Table 8:** In-lab transitions, the recorded transitions, the difference and the percentage difference.

Transitions	Planned Transitions	Recorded Transitions	Difference	% Difference
Standing-transition-sitting	380	374	−6	−1.58%
Sitting-transition-standing	380	309	−71	−18.68%
Sitting-transition-picking	180	182	2	1.11%
Picking-transition-sitting	180	164	−16	−8.89%
Walking-transition-picking	180	166	−14	−7.78%
Shuffling-transition-picking		7	n/a	
Standing-transition-picking	60	66	6	10.00%
Picking-transition-walking	180	142	−38	−21.11%
Picking-transition-shuffling		32	n/a	
Picking-transition-standing	60	82	22	36.67%
Sitting-transition-walking	120	150	30	25.00%
Sitting-transition-shuffling		45	n/a	
Walking-transition-sitting	120	99	−21	−17.50%
Shuffling-transition-sitting		55	n/a	
Sitting-transition-lying	60	76	16	26.67%
Lying-transition-sitting	60	66	6	10.00%
Lying-transition-walking	60	74	14	23.33%
Lying-transition-shuffling		17	n/a	
Walking-transition-lying	60	33	−27	−45.00%
Shuffling-transition-lying		49	n/a	
Standing-transition-leaning	60	65	5	8.33%
Walking-transition-leaning		7	n/a	
Sitting-transition-leaning		3	n/a	
Shuffling-transition-leaning		1	n/a	
Leaning-transition-standing	60	65	5	8.33%
Leaning-transition-walking		7	n/a	
Leaning-transition-sitting		3	n/a	
Leaning-transition-leaning		2	n/a	
Leaning-transition-shuffling		1	n/a	
Standing-transition-lying	100	62	−38	−38.00%
Lying-transition-standing	100	51	−49	−49.00%
Sitting-transition-kneeling	60	30	−30	−50.00%
Kneeling-transition-sitting	60	22	−38	−63.33%
Kneeling-transition-standing	60	30	−30	−50.00%
Kneeling-transition-shuffling		4	n/a	
Standing-transition-kneeling	60	25	−35	−58.33%
Sitting-transition-sitting		85	n/a	
Lying-transition-lying		12	n/a	
Standing-transition-standing		8	n/a	
Picking-transition-picking		6	n/a	
Total	2640	2677	37	

**Table 9:** Inter-rater reliability statistics for the out-of-lab scenario.

Inter-Rater Reliability Statistics	Average	Maximum	Minimum
Category agreement (%)	90.05	93.31	87.93
Cohen’s kappa	0.86	0.91	0.83
Corrected kappa	0.89	0.93	0.87
Krippendorff’s alpha	0.86	0.91	0.83

**Table 10:** A summary of the out-of-lab activities, excluding Undefined, Static, Dynamic, Shake and Jumping, which were used for synchronisation.

Activities	Quantity	Maximum Bout (s)	Minimum Bout (s)	Average Bout (s)	Standard Deviation (s)	Total (s)	Total (%)
Sitting	576	2075.6	0.04	103.42	221.04	59568	48.09%
Standing	4837	388.52	0	5.68	12.26	27458	22.17%
Walking	2926	163.6	0.28	6.02	9.91	17617	14.22%
Transition	3454	35.76	0.24	1.84	1.64	6346	5.12%
Shuffling	4290	20	0.04	1.35	1.31	5780	4.67%
Leaning	1233	67	0.0399	2.33	5.31	2870	2.32%
Lying	14	583.8	3.48	117.48	144.54	1645	1.33%
Stairs (ascending)	152	20.24	1.32	7.10	3.17	1079	0.87%
Stairs (descending)	120	17.48	1.1599	6.67	3.24	801	0.65%
Picking	306	29.84	0.033	2.25	4.10	688	0.56%
Kneeling	2	14.04	5.4	9.72	6.11	19	0.02%
					Total (s)	123,870.130	
					Total (h)	34.408	

**Table 11:** Activity-Transition-Activity quantity.

Transitions	Planned Transitions	Recorded Transitions	Difference	% Difference
Standing-transition-leaning	100	271	171	171%
Standing-transition-lying	20	1	−19	−95.0%
Standing-transition-sitting	120	30	−90	−75.0%
Standing-transition-picking	40	68	28	70.0%
Sitting-transition-standing	120	37	−83	−69.17%
Sitting-transition-walking	120	115	−5	−4.17%
Sitting-transition-leaning		152	n/a	
Sitting-transition-lying	20	8	−12	−60.0%
Sitting-transition-shuffling		39	n/a	
Sitting-transition-sitting		187	n/a	
Lying-transition-sitting	20	9	−11	−55.0%
Lying-transition-walking	20	4	−16	−80.0%
Lying-transition-standing	20	0	−20	−100.0%
Leaning-transition-standing	80	312	232	290.0%
Leaning-transition-walking	100	241	141	141.0%
Leaning-transition-sitting		151	n/a	
Leaning-transition-shuffling		286	n/a	
Leaning-transition-leaning		174	n/a	
Picking-transition-standing	40	83	43	107.5%
Walking-transition-sitting	120	74	−46	−38.33%
Walking-transition-leaning	100	325	225	225.0%
Walking-transition-lying	20	3	−17	−85.0%
Shuffling-transition-leaning		264	n/a	
Shuffling-transition-sitting		104	n/a	
Shuffling-transition-picking		91	n/a	
Picking-transition-shuffling		86	n/a	
Walking-transition-picking		80	n/a	
Picking-transition-walking		74	n/a	
Leaning-transition-picking		29	n/a	
Picking-transition-leaning		26	n/a	
Leaning-transition-undefined		25	n/a	
Picking-transition-picking		19	n/a	
Undefined-transition-leaning		17	n/a	
Sitting-transition-picking		11	n/a	
Picking-transition-sitting		8	n/a	
Picking-transition-undefined		7	n/a	
Undefined-transition-picking		5	n/a	
Sitting-transition-undefined		4	n/a	
Undefined-transition-shuffling		3	n/a	
Undefined-transition-sitting		3	n/a	
Shuffling-transition-shuffling		2	n/a	
Shuffling-transition-lying		2	n/a	
Picking-transition-kneeling		2	n/a	
Undefined-transition-walking		2	n/a	
Stairs (ascending)-transition-leaning		1	n/a	
Leaning-transition-stairs (ascending)		1	n/a	
Stairs (ascending)-transition-picking		1	n/a	
Shuffling-transition-undefined		1	n/a	
Lying-transition-shuffling		1	n/a	
Kneeling-transition-walking		1	n/a	
Kneeling-transition-standing		1	n/a	
Stairs (descending)-transition-picking		1	n/a	
Total	1080	3442		

**Table 12:** Summary of the in-lab and out-of-lab activities, excluding Undefined, Static, Dynamic, Shake and Jumping.

Activities	Quantity	Maximum Bout (s)	Minimum Bout (s)	Average Bout (s)	Standard Deviation (s)	Total (s)	Total (%)
Sitting	1461	2075.6	0.033	46.33	147.01	67,681	42.80%
Standing	6455	388.52	0.033	6.06	13.42	39,116	24.73%
Walking	3706	163.6	0.04	5.96	8.93	22,079.7	13.96%
Transition	6146	35.76	0.234	2.05	1.56	12,623.8	7.98%
Shuffling	5402	36.266	0.033	1.46	1.55	7870.8	4.98%
Lying	246	583.8	0.133	12.39	43.23	3047.8	1.93%
Leaning	1311	67	0.033	2.22	5.17	2909.3	1.84%
Stairs (ascending)	152	20.24	1.32	7.10	3.17	1078.5	0.68%
Stairs (descending)	120	17.48	1.1599	6.67	3.24	800.6	0.51%
Picking	733	29.84	0.0329	1.07	2.84	786.4	0.50%
Kneeling	58	33.84	0.067	2.63	5.15	153	0.10%
					Total (s)	158,146.9	
					Total (h)	43.9297	

## Appendix A

**Table A1:** Postures, transitions and behaviours for the supervised semi-structured protocol for each task on a per subject basis and for a total of 20 subjects, assuming each task is repeated 3 times.

**Per 20 Subjects**		**380**	**380**	**100**	**100**	**60**	**60**	**60**	**60**	**60**	**60**	
**Per Subject**		**19**	**19**	**5**	**5**	**3**	**3**	**3**	**3**	**3**	**3**	
	**Repeat**	**Sit-to-Stand**	**Stand-to-Sit**	**Stand-to-Lie**	**Lie-to-Stand**	**Sit-to-Lie**	**Lie-to-Sit**	**Stand-to-Kneel**	**Kneel-to-Stand**	**Stand-to-Lean**	**Lean-to-Stand**	
Stand-sit-stand at a table	3	3	3									
Stand-sit-stand on a soft chair	3	3	3									
Sit-kneel-sit	3											
Stand-lie-stand	3			3	3							
Sit-lie-sit	3			1	1	3	3					
Stand-kneel-stand	3							3	3			
Stand-pick and object off the floor-stand	3											
Stand and lean to pick and object of a table forward-stand	3									3	3	
Stand-sit, while sitting, pick an object off the floor forward, stand	3	3	3									
Stand-sit, while sitting, pick an object off the floor right stand	3	3	3									
Stand-sit, while sitting, pick an object off the floor left, stand	3	3	3									
Stand-sit at a table-walk-pick an object-sit-stand	3	3	3									
Stand-lie-walk-pick an object-lie-stand	3			1	1							
Sit on a soft chair-walk-pick an object-walk-sit	3	1	1									
Stand-move objects from one table to the other	3											
Stand-walk(normal)-stand	3											
Stand-walk(fast)-stand	3											
Stand-walk(slow)-stand	3											
Stand-climb stairs-stand-descend-stairs	3											
**Per 20 Subjects**		**540**	**540**	**180**	**180**	**120**	**120**	**60**	**60**	**0**	**0**	
**Per Subject**		**27**	**27**	**9**	**9**	**6**	**6**	**3**	**3**	**0**	**0**	
		**Stand-to-Walk**	**Walk-to-Stand**	**Sitting-to-Picking**	**Picking-to-Sitting**	**Sit-to-Walk**	**Walk-to-Sit**	**Lie-to-Walk**	**Walk-to-Lie**	**Walk-to-Kneel**	**Kneel-to-Walk**	
Stand-sit-stand at a table												
Stand-sit-stand on a soft chair												
Sit-kneel-sit												
Stand-lie-stand												
Sit-lie-sit												
Stand-kneel-stand												
Stand-pick and object off the floor-stand												
Stand and lean to pick and object of a table forward-stand												
Stand-sit, while sitting, pick an object off the floor forward, stand				3	3							
Stand-sit, while sitting, pick an object off the floor right stand				3	3							
Stand-sit, while sitting, pick an object off the floor left, stand				3	3							
Stand-sit at a table-walk-pick an object-sit-stand		3	3			3	3					
Stand-lie-walk-pick an object-lie-stand								3	3			
Sit on a soft chair-walk-pick an object-walk-sit						3	3					
Stand-move objects from one table to the other		3	3									
Stand-walk(normal)-stand		6	6									
Stand-walk(fast)-stand		6	6									
Stand-walk(slow)-stand		6	6									
Stand-climb stairs-stand-descend-stairs		3	3									
**Per 20 Subjects**		**180**			**180**		**60**		**60**		**0**	**0**
**Per Subject**		**9**			**9**		**3**		**3**		**0**	**0**
		**Walking-to-Picking**			**Picking-to-Walking**		**Stand-to-Picking**		**Picking-to-Stand**		**Lean-to-Walk**	**Walk-to-Lean**
Stand-sit-stand at a table												
Stand-sit-stand on a soft chair												
Sit-kneel-sit												
Stand-lie-stand												
Sit-lie-sit												
Stand-kneel-stand												
Stand-pick and object off the floor-stand							3		3			
Stand and lean to pick and object of a table forward-stand												
Stand-sit, while sitting, pick an object off the floor forward, stand												
Stand-sit, while sitting, pick an object off the floor right stand												
Stand-sit, while sitting, pick an object off the floor left, stand												
Stand-sit at a table-walk-pick an object-sit-stand		3			3							
Stand-lie-walk-pick an object-lie-stand		3			3							
Sit on a soft chair-walk-pick an object-walk-sit		3			3							
Stand-move objects from one table to the other												
Stand-walk(normal)-stand												
Stand-walk(fast)-stand												
Stand-walk(slow)-stand												
Stand-climb stairs-stand-descend-stairs												
**Per 20 Subjects**		**60**			**60**		**60**		**60**	**60**	**60**	**60**
**Per Subject**		**3**			**3**		**3**		**3**	**3**	**3**	**3**
		**Sit-to-Kneel**			**Kneel-to-Sit**		**Climb Stairs-to-Stand**		**Descend Stairs-to-Stand**	**Stand-to-Climb Stairs**	**Stand-to-Descend Stairs**	**Shuffling**
Stand-sit-stand at a table												
Stand-sit-stand on a soft chair												
Sit-kneel-sit		3			3							
Stand-lie-stand												
Sit-lie-sit												
Stand-kneel-stand												
Stand-pick an object off the floor-stand												
Stand and lean to pick and object of a table forward-stand												
Stand-sit, while sitting, pick an object off the floor forward, stand												
Stand-sit, while sitting, pick an object off the floor right stand												
Stand-sit, while sitting, pick an object off the floor left, stand												
Stand-sit at a table-walk-pick an object-sit-stand												
Stand-lie-walk-pick an object-lie-stand												
Sit on a soft chair-walk-pick an object-walk-sit												
Stand-move objects from one table to the other												3
Stand-walk(normal)-stand												
Stand-walk(fast)-stand												
Stand-walk(slow)-stand												
Stand-climb stairs-stand-descend-stairs							3		3	3	3	
**Per 20 Subjects**		**1080**		**540**	**180**	**120**	**240**	**240**	**780**	**60**	**60**	
**Per Subject**		**54**		**27**	**9**	**6**	**12**	**12**	**39**	**3**	**3**	
		**Standing**		**Sitting**	**Lying**	**Kneeling**	**Squat Down Posture (Object Picking**	**Leaning Posture to Each Side**	**Walking**	**Ascending-Stairs**	**Descend-Stairs**	
Stand-sit-stand at a table		3		3					3			
Stand-sit-stand on a soft chair		3		3					3			
Sit-kneel-sit				3		3						
Stand-lie-stand		3			3							
Sit-lie-sit		3		3	3							
Stand-kneel-stand		3				3						
Stand-pick an object off the floor-stand		3					3		6			
Stand and lean to pick and object of a table forward-stand		3						3				
Stand-sit, while sitting, pick an object off the floor forward, stand		3		3				3				
Stand-sit, while sitting, pick an object off the floor right stand		3		3				3				
Stand-sit, while sitting, pick an object off the floor left, stand		3		3				3				
Stand-sit at a table-walk-pick an object-sit-stand		3		3			3		3			
Stand-lie-walk-pick an object-lie-stand		3			3		3		3			
Sit on a soft chair-walk-pick an object-walk-sit		3		3			3		3			
Stand-move objects from one table to the other		3							3			
Stand-walk(normal)-stand		3							3			
Stand-walk(fast)-stand		3							3			
Stand-walk(slow)-stand		3							3			
Stand-climb stairs-stand-descend-stairs		3							6	3	3	

## Appendix B

**Table A2:** Postures, transitions and behaviours for the free-living protocol for each task on a per subject basis and for a total of 20 subjects, assuming each task is repeated once.

**Per 20 Subjects**		**120**	**120**	**20**	**20**	**20**	**20**		**100**	**100**	**60**	**60**
**Per Subject**		**6**	**6**	**1**	**1**	**1**	**1**		**5**	**5**	**3**	**3**
	**Repeat**	**Sit-to-Stand**	**Stand-to-Sit**	**Stand-to-Lie**	**Lie-to-Stand**	**Sit-to-Lie**	**Lie-to-Sit**		**Stand-to-Lean**	**Lean-to-Stand**	**Stand-to-Walk**	**Walk-to-Stand**
Sit at a table and write a letter/list or read	**1**	1	1									
Sit on an armchair watch TV, video or read a magazine	**1**	1	1									
Sit on a low stool or toilet seat (lid down clothes on, simulation only)	**1**	1	1									
Lie on a bed, clothes on (CBF10)	**1**			1	1	1	1					
Get in and out of a car (CBF10) or sitting on a bed	**1**	1	1									
Prepare and consume a drink or food while standing	**1**								1	1		
Set a table for dinner or move from one counter to another many times (up to 10) (shuffling)	**1**										1	1
Simulate unloading a washing machine for 10 s or preparing a fireplace	**1**								1	1		
Pick an object of the floor/replace or tie or untie shoe laces	**1**											
Climbing and descending stairs or walking up and down an inclined path	**1**											
Remove clothes from washing machine and hang on clothes rack or remove rubbish from bin and dispose	**1**								1	1		
Sit and prepare and eat something	**1**	1	1									
Clean mirror or clean a window	**1**								1	1	1	1
Wash and dry hands	**1**								1	1	1	1
Sit at a table and read	**1**	1	1									
**Per 20 Subjects**		**120**	**120**	**20**	**20**	**40**	**40**		**100**		**100**	
**Per Subject**		**6**	**6**	**1**	**1**	**2**	**2**		**5**		**5**	
		**Sit-to-Walk**	**Walk-to-Sit**	**Lie-to-Walk**	**Walk-to-Lie**	**Squat down-to-Pick an Object**	**Standing up after Picking up an Object**		**Lean-to-Walk**		**Walk-to-Lean**	
Sit at a table and write a letter/list or read		1	1									
Sit on an armchair watch TV, video or read a magazine		1	1									
Sit on a low stool or toilet seat (lid down clothes on, simulation only)		1	1									
Lie on a bed, clothes on (CBF10)				1	1							
Get in and out of a car (CBF10) or sitting on a bed		1	1									
Prepare and consume a drink or food while standing									1		1	
Set a table for dinner or move from one counter to another many times (up to 10) (shuffling)						1	1					
Simulate unloading a washing machine for 10 s or preparing a fireplace									1		1	
Pick an object of the floor/replace or tie or untie shoe laces						1	1		1		1	
Climbing and descending stairs or walking up and down an inclined path												
Remove clothes from washing machine and hang on clothes rack or remove rubbish from bin and dispose												
Sit and prepare and eat something		1	1									
Clean mirror or clean a window									1		1	
Wash and dry hands									1		1	
Sit at a table and read		1	1									
**Per 20 Subjects**		**20**	**20**	**40**	**680**	**520**	**120**	**80**	**400**		**600**	
**Per Subject**		**1**	**1**	**2**	**34**	**26**	**6**	**4**	**20**		**30**	
		**Ascending Stairs**	**Descend-Stairs**	**Shuffling**	**Standing**	**Sitting**	**Lying**	**Squat down Posture (Object Picking)**	**Leaning Posture to Each Side**		**Walking**	
Sit at a table and write a letter/list or read					2	4					2	
Sit on an armchair watch TV, video or read a magazine					2	4					2	
Sit on a low stool or toilet seat (lid down clothes on, simulation only)					2	4					2	
Lie on a bed, clothes on (CBF10)					2	2	6				2	
Get in and out of a car (CBF10) or sitting on a bed					2	4					2	
Prepare and consume a drink or food while standing					2				4		2	
Set a table for dinner or move from one counter to another many times (up to 10) (shuffling)				2	4			2			2	
Simulate unloading a washing machine for 10 s or preparing a fireplace					2			0	4		2	
Pick an object of the floor/replace or tie or untie shoe laces					2			2	2		2	
Climbing and descending stairs or walking up and down an inclined path		1	1									
Remove clothes from washing machine and hang on clothes rack or remove rubbish from bin and dispose					2				2			
Sit and prepare and eat something					2	4					2	
Clean mirror or clean a window					4				4		4	
Wash and dry hands					4				4		4	
Sit at a table and read					2	4					2	

## Appendix C

**Table A3:** The instruction set for the semi-structured task-based protocol.

			Start Posture	End Posture
*1*	**Stand on A and sit at the table, repeat**	**Zone 1**		
	Stand on A		stand	stand
	Sit at the table		stand	sitting
	Stand on A		sitting	stand
	Sit at the table		stand	sitting
	Stand on A		sitting	stand
	Sit at the table		stand	sitting
	Stands on A		sitting	stand
*2*	**Stand-sit at a table-walk-pick an object-sit-stand**	**Zone 1**		
	Stand on A		stand	stand
	Sit at the table beside A		stand	sitting
	Walk and touch F with your hand and then sit at the table beside A		sitting	sitting
	Walk and touch F with your hand and then sit at the table beside A		sitting	sitting
	Walk and touch F with your hand and then sit at the table beside A		sitting	sitting
	Stand on A		sitting	stand
*3*	**Stand and lean to pick and object of a table FW-stand**	**Zone 1**		
	Stand on A		stand	stand
	Lean and touch G and then return to standing		stand	stand
	Lean and touch G and then return to standing		stand	stand
	Lean and touch G and then return to standing		stand	stand
	Walk and stand on E		stand	stand
*4*	**Stand-pick and object off the floor-stand**	**Zone 2**		
	Stand on E		stand	stand
	Bend down and touch E and return to standing		stand	stand
	Bend down and touch E and return to standing		stand	stand
	Bend down and touch E and return to standing		stand	stand
	Stand on E		stand	stand
*5*	**Lean and pick objects while sitting**	**Zone 2**		
	Stand on E		stand	stand
	Sit on the chair		stand	sitting
	While sitting, pick an object off the floor forward		sitting	sitting
	Stand on E		sitting	stand
	Sit on the chair		stand	sitting
	While sitting, pick an object off the floor left		sitting	sitting
	Stand on E		sitting	stand
	Sit on the chair		stand	sitting
	While sitting, pick an object off the floor right		sitting	sitting
	Stand on E		sitting	stand
	Sit on the chair		stand	sitting
	While sitting, pick an object off the floor forward		sitting	sitting
	Stand on E		sitting	stand
	Sit on the chair		stand	sitting
	While sitting, pick an object off the floor left		sitting	sitting
	Stand on E		sitting	stand
	Sit on the chair		stand	sitting
	While sitting, pick an object off the floor right		sitting	sitting
	Stand on E		sitting	stand
	Sit on the chair		stand	sitting
	While sitting, pick an object off the floor forward		sitting	sitting
	Stand on E		sitting	stand
	Sit on the chair		stand	sitting
	While sitting, pick an object off the floor left		sitting	sitting
	Stand on E		sitting	stand
	Sit on the chair		stand	sitting
	While sitting, pick an object off the floor right		sitting	sitting
	Stand on E		sitting	stand
	Walk to B and stand		stand	stand
*6*	**Stand-sit on a soft chair-stand**	**Zone 3**		
	Stand on B		stand	stand
	Sit on the arm chair		stand	sitting
	Stand on B		sitting	stand
	Sit on the arm chair		stand	sitting
	Stand on B		sitting	stand
	Sit on the arm chair		stand	sitting
	Stand on B		sitting	stand
*7*	**Stand-sit-walk-pick an object-walk-sit-stand**	**Zone 3**		
	Stand on B		stand	stand
	Sit on an armchair		stand	sitting
	Walk and touch F and then sit on an armchair		sitting	sitting
	Walk and touch F and then sit on an armchair		sitting	sitting
	Walk and touch F and then sit on an armchair		sitting	sitting
	Walk and stand at C		sitting	stand
*8*	**Sit-kneel-sit**	**Zone 4**		
	Stand at C		stand	stand
	Sit on the chair		stand	sitting
	Kneel on the cushion		sitting	kneel
	Sit on the chair		kneel	sitting
	Kneel on the cushion		sitting	kneel
	Sit on the chair		kneel	sitting
	Kneel on the cushion		sitting	kneel
	Sit on the chair		kneel	sitting
	Stand at C		stand	stand
	Walk and stand on D		walk	stand
***9***	**Stand-lie-stand**	**Zone 4**		
	Stand on D		stand	stand
	Lie on the bed		stand	lie
	Stand on D		lie	stand
	Lie on the bed		stand	lie
	Stand on D		lie	stand
	Lie on the bed		stand	lie
	Stand on D		lie	stand
*10*	**Sit-lie-sit**	**Zone 4**		
	Sit on the bed		stand	sit
	Lie on the bed		sitting	lie
	Sit on the bed		lie	sitting
	Lie on the bed		sitting	lie
	Sit on the bed		lie	sitting
	Lie on the bed		sitting	lie
	Sit on the bed		lie	sitting
	Stand on D		sitting	stand
***11***	**Stand-kneel-stand**	**Zone 4**		
	Stand on D		stand	stand
	Kneel on the cushion		stand	kneel
	Stand on D		kneel	stand
	Kneel on the cushion		stand	kneel
	Stand on D		kneel	stand
	Kneel on the cushion		stand	kneel
	Stand on D		kneel	stand
	Walk to D and stand		stand	stand
***12***	**Stand-lie-walk-pick an object-lie-stand**	**Zone 4**		
	Stand on D		stand	stand
	Lie on the bed		stand	lie
	Walk and touch E return and lie on the bed		lie	lie
	Walk and touch E return and lie on the bed		lie	lie
	Walk and touch E return and lie on the bed		lie	lie
	Walk and stand on A		lie	stand
***13***	**Stand-move objects from one table to the other**	**Zone 1**		
	Stands on A		stand	stand
	Move objects from one table to the other		stand	stand
	Stand on A		stand	stand
	Move objects from one table to the other		stand	stand
	Stand on A		stand	stand
	Move objects from one table to the other		stand	stand
	Stand on A		stand	stand
***14***	**Stand-walk(normal)-stand**	**Zone 2**		
	Stand on A		stand	stand
	Walk at normal speed to F		stand	stand
	Stand on A		stand	stand
	Walk at normal speed to F		stand	stand
	Stand on A		stand	stand
	Walk at normal speed to F		stand	stand
	Stand on A		stand	stand
***15***	**Stand-walk(fast)-stand**	**Zone 2**		
	Stand on A		stand	stand
	Walk at fast speed to F		stand	stand
	Stand on A		stand	stand
	Walk at fast speed to F		stand	stand
	Stand on A		stand	stand
	Walk at fast speed to F		stand	stand
	Stand on A		stand	stand
***16***	**Stand-walk(slow)-stand**	**Zone 2**		
	Stand on A		stand	stand
	Walk at slow speed to F		stand	stand
	Stand on A		stand	stand
	Walk at slow speed to F		stand	stand
	Stand on A		stand	stand
	Walk at slow speed to F		stand	stand
	Stand on A		stand	stand
	**Attach and sync the GoPro with a lying movement and a jump and call the taxi**			
	Walk and stand at the bottom of the stairs		stand	stand
*17*	**Stand-climb stairs-stand-descend stairs**	**Out-of-lab**		
	Stand at the bottom of the stairs		stand	stand
	Walk up stairs		stand	stand
	Stand		stand	stand
	Salk down stairs		stand	stand
	Stand		stand	stand
	Walk up stairs		stand	stand
	Stand		stand	stand
	Walk down stairs		stand	stand
	Stand		stand	stand
	Walk up stairs		stand	stand
	Stand		stand	stand
	Walk down stairs		stand	stand
	Stand		stand	stand
	Continue with the out-of-lab scenario			
